# Longitudinal effects of inpatient music therapy dosage on pain intensity and opioid utilization among patients with hematologic or oncologic conditions: a retrospective cohort study

**DOI:** 10.1007/s00520-026-10865-x

**Published:** 2026-06-06

**Authors:** Samuel N. Rodgers-Melnick, Douglas Gunzler, Thomas E. Love, Siran M. Koroukian, Mark Beno, Santosh Rao, Rab Razzak, Jeffery A. Dusek, Johnie Rose

**Affiliations:** 1 Connor Whole Health, University Hospitals of Cleveland, 11100 Euclid Avenue, Cleveland, OH 44106 USA; 2 Department of Population and Quantitative Health Sciences, Case Western Reserve University School of Medicine, Cleveland, OH USA; 3 Department of Psychiatry, Case Western Reserve University School of Medicine, 11100 Euclid Avenue, Cleveland, OH 44106 USA; 4 Population Health Research Institute, The MetroHealth System, Cleveland, OH USA; 5 Department of Medicine, Case Western Reserve University School of Medicine, Cleveland, OH USA; 6 Cleveland Institute for Computational Biology, Case Western Reserve University School of Medicine, Cleveland, OH USA; 7 Case Comprehensive Cancer Center, Case Western Reserve University School of Medicine, Cleveland, OH USA; 8 Sutter West Bay Medical Group, San Francisco, CA USA; 9 Division of Palliative Care, California Pacific Medical Center, San Francisco, CA USA; 10 Department of Medicine, University of California – Irvine, Irvine, CA USA; 11 Susan Samueli Integrative Health Institute, University of California – Irvine, Irvine, CA USA; 12 Center for Community Health Integration, Case Western Reserve University School of Medicine, Cleveland, OH USA

**Keywords:** Cancer, Music therapy, Oncology, Opioids, Pain, Sickle cell disease

## Abstract

**Purpose:**

Several studies support the benefits of music therapy (MT) for improving symptoms within a single session among patients with hematologic/oncologic conditions, but none have examined real-world longitudinal impacts. This study evaluated whether there was a dose–response relationship between MT intervention exposure and longitudinal changes in pain intensity and opioid utilization.

**Methods:**

A retrospective electronic health record review was conducted of 283 hospital admissions among adult patients with hematologic/oncologic conditions who received MT and either reported pain ≥ 4/10 on the numeric rating scale (NRS) or received opioids in the first 48 h of admission. Longitudinal changes in pain intensity on the 0–10 NRS and log-transformed morphine milligram equivalents were modeled using linear mixed-effects models with natural splines for time since first MT intervention interacting with MT exposure group (1, 2, or ≥ 3 MT interventions). Models were adjusted for baseline values and length of stay.

**Results:**

Of 283 hospital admissions, 122 (43.1%) included 1, 89 (31.4%) included 2, and 72 (25.4%) included ≥ 3 MT interventions. No meaningful MT exposure-by-time effects were found for pain intensity (*p* = .131) or opioid utilization per day (*p* = .118).

**Conclusions:**

Among patients admitted with hematologic/oncologic conditions, greater MT exposure does not appear to reduce pain intensity or opioid exposure over time relative to receiving 1 MT intervention. Future evaluations of inpatient programs should consider collecting more robust data on outcomes that may be more sensitive to intervention (e.g., stress, anxiety) and increasing the MT dose to have a greater impact on outcomes over time.

## Background

Hospital admissions related to cancer and/or hematologic conditions such as sickle cell disease (SCD) are often complicated by multiple co-occurring symptoms including pain, anxiety, nausea, and fatigue. Despite growing recognition of pain’s prevalence and intensity within this population, many patients still report inadequate analgesia. A systematic review of 66 studies among adults with cancer and pain estimated that 40% of patients report inadequate analgesic treatment on the pain management index [32]. Patients with SCD face unique pain management challenges due to (1) complex and poorly understood pathophysiology (e.g., vaso-occlusion, inflammation, increased red blood cell adhesion, central sensitization, and opioid-induced hyperalgesia) underlying both acute and chronic pain [9]; (2) frequent experiences with discrimination [25]; (3) poor patient-provider communication [17] in the healthcare system; and (4) structural barriers in accessing timely and appropriate care [29]. When suboptimal pain management persists, patients may experience prolonged psychological distress and impairment following hospital discharge [18].

While health systems caring for patients with hematologic and/or oncologic conditions are obligated to address patients’ acute pain, this can be extremely challenging given the risks and adverse events (e.g., pruritus, nausea, constipation, sedation, respiratory depression) associated with opioids and other analgesic medications [18]. Given directives from the Joint Commission to promote and provide nonpharmacologic modalities [41] and the American Society of Hematology’s recommendation to integrate nonpharmacologic interventions within SCD pain management [9], many cancer centers are now making an intentional shift to providing evidence-based integrative modalities [12].

Music therapy (MT) is defined as the clinical use of tailored music interventions (e.g., active music making, music-assisted relaxation and imagery, and songwriting) to accomplish individualized goals within a therapeutic relationship by a credentialed professional (i.e., a board-certified music therapist) [1]. Several studies have demonstrated MT’s efficacy for addressing acute pain among patients with hematologic and/or oncologic conditions [6]. Importantly, the therapeutic relationship between the music therapist and the patient makes MT distinct from other music-based interventions such as music medicine where healthcare professionals such as nurses provide patients with recorded music interventions [6]. A systematic review of 81 music intervention (38 MT and 43 music medicine) trials with 5,576 participants found that music interventions may have positive effects on pain, anxiety, depression, hope, and fatigue among adults with cancer as compared to standard care [6]. Prior studies also support MT’s analgesic benefits among adults with SCD [37].

MT has also demonstrated efficacy for reducing opioid exposure among patients with cancer. An RCT comparing two live MT sessions to standard supportive care among 82 patients with lymphoma or multiple myeloma undergoing autologous stem cell transplantation found no meaningful group-by-time effect in pain scores (*p* = 0.19), but patients who received MT used less opioid pain medication (median, 24 mg versus 73 mg; *p* = 0.038) from day −1 to day + 5 compared to controls [3]. In a recent pilot study among 26 cancer survivors with chronic pain and persistent opioid use, participants were randomized to either 10 weekly, individual interactive MT sessions (*n* = 14) or social attention control (*n* = 12) sessions. The researchers found a large effect for interactive MT on reducing mean daily opioid use at week 10 and at 3-month follow-up [8].

Many cancer centers have also begun to investigate MT’s real-world clinical effectiveness within a single session. Specifically, a retrospective study among 96 inpatients at an academic cancer center found that patients reported clinically significant mean improvements in Edmonton Symptom Assessment Scale (ESAS) 0–10 numeric scores including pain (−1.8 units), anxiety (−2.3 units), and fatigue (−1.9 units) [23]. In our prior study examining 4,002 sessions among 1,152 patients treated at an academic cancer center, reductions in ESAS measures of pain (−1.48 units), anxiety (−2.58 units) and fatigue (−0.84) units were observed in the combined sample, with changes in pain and anxiety exceeding clinically significant (i.e., ≥ 1 unit [24]) thresholds [38].

However, to date no study has examined the real-world impact of MT on longitudinal measures of pain intensity or opioid utilization over the course of a hospital admission among patients with hematologic and/or oncologic conditions. To address this gap, the purpose of the current study was to determine if there is a dose–response relationship between the number of MT interventions provided and longitudinal changes in the following outcomes measured from the time of first MT intervention until 144 h following the first MT intervention: (1) pain intensity on the 0–10 numeric rating scale (NRS) as collected by nurses and (2) morphine milligram equivalents (MME) administered per 24-h period.

## Methods

### Participants and design

This study is a retrospective electronic health record (EHR) review of 283 hospital admissions ≥ 120 h across 10 hospitals among adult patients with hematologic/oncologic conditions meeting the following criteria: (1) admitted between August 2020 and August 2023; (2) received ≥ 1 MT intervention during their hospital admission; (3) had a primary or secondary diagnosis of a neoplasm (International Classification of Diseases [ICD]−10 code C00 – D49) or sickle cell disease (ICD-10 code D57) associated with their hospital admission; (4) rated their pain ≥ 4/10 on the NRS or received opioids in the first 48 h of their hospital admission; (5) received or were referred to MT < 50% of the way through their hospitalization; (6) had pain NRS scores collected within the 24 h prior to the first MT intervention; (7) had a mean number of pain NRS scores collected per day after the first MT intervention ≥ 2; (8) had ≥ 5 total pain NRS scores collected after the first MT intervention; and (9) were discharged 3–8 days after the first MT intervention. Patients who were admitted to an inpatient psychiatry floor, discharged to hospice, seen by a hospice professional during their admission, or noted as deceased in their discharge summaries were excluded from analysis.

### Setting and care delivery

MT interventions were provided across ten medical centers within University Hospitals (UH), a non-profit health system in Northeast Ohio serving more than 1.2 million patients per year. MT services within the UH system are provided without cost to patients (i.e., not billed to insurance) and funded through multiple sources including foundation grants, philanthropy, and each hospital’s operating budget. Music therapists are integrated within clinical care and collaborate with other healthcare professionals (e.g., physicians, advanced practice providers, nurses, social workers, chaplains, etc.) to address patients’ symptoms and enhance psychosocial support. MT’s integration within the freestanding academic cancer center at UH, including inpatient, infusion, radiation, and outpatient areas, has been described previously [38].

### Ethics and permissions

This study was approved by the UH Cleveland Medical Center Institutional Review Board as a retrospective chart review (STUDY20191213) with a waiver of informed consent in accordance with the Declaration of Helsinki.

### Data collected

All data were extracted from the UH Allscripts EHR and Electronic Data Warehouse (EDW) using multiple structured query language (SQL) scripts. These data included (1) socio-demographic information including age, sex, race/ethnicity, marital status, insurance status, and social vulnerability index (SVI); (2) clinical characteristics including Elixhauser comorbidity count [13] and presence of key pain-related diagnoses based on ICD-10 codes for SCD, neoplasms, and mental health/substance use disorders (MSUD); (3) patients’ total opioid exposure during their hospital admissions, including dosage, drug name, route, and administration datetime; (4) all pain NRS scores collected by nurses or music therapists over the course of the hospital admission and their associated datetimes; and (5) MT intervention characteristics including intervention category and music therapist name. MME administered per day were calculated after curating data on oral, intravenous, patch, and patient-controlled analgesia exposures and converting to oral MME using guidance from McPherson [26].

### Data analysis

Baseline differences between the three exposure groups (1, 2, or ≥ 3 MT interventions) were examined using Kruskal–Wallis rank sum tests, Pearson’s Chi-squared tests, and Fisher’s exact tests. Linear mixed effects models were initially fit without nonlinear terms (i.e., “Linear time model”) to examine exposure-by-time effects on longitudinal outcomes after accounting for baseline outcome measures, random music therapist effects, and length of stay (hours). Linear mixed effects models are well-suited for this examination as they can accommodate heterogeneity in timing and completion of data collection to calculate the slope trajectory of NRS scores and MME administered per day over the course of patients’ hospital admissions. A linear mixed effects approach is also well-suited for handling correlated repeated measurements, unbalanced data, missing data, and confounding in longitudinal studies [14].

For the pain model, time was defined as the distance (in hours) between the time of the first MT intervention and the recorded pain score. For the MME model, MME/day outcome measures were quantified within 24-h periods relative to the first MT intervention and log-transformed. Given the limited availability of longitudinal outcome data within each exposure group beyond 144 h in the pain model (18.0% of 1 MT intervention group, 21.3% of 2 MT interventions group, and 51.4% of ≥ 3 MT interventions group available after 144 h) and the opioid model (42.6% of 1 MT intervention group, 53.9% of 2 MT interventions group, and 73.6% of ≥ 3 MT interventions group available after 144 h), longitudinal data were only included in the models up until 144 h following the first MT intervention. To account for observed non-linearity in response trajectories, an additional series of models was fit with a natural spline of time since first MT intervention (df = 3) (i.e., “Spline model”). Analysis of variance (ANOVA) was applied to both the Linear time and Spline models to test whether outcome trajectories differed over time across the MT exposure groups. All linear mixed effects models were fit using the lmer function from the “lme4” package [4]. All analyses and plots were generated using R Version 4.5.2 [30] and RStudio Version 2026.01.0 + 392 [31].

## Results

### Sample characteristics

**Fig. 1:**
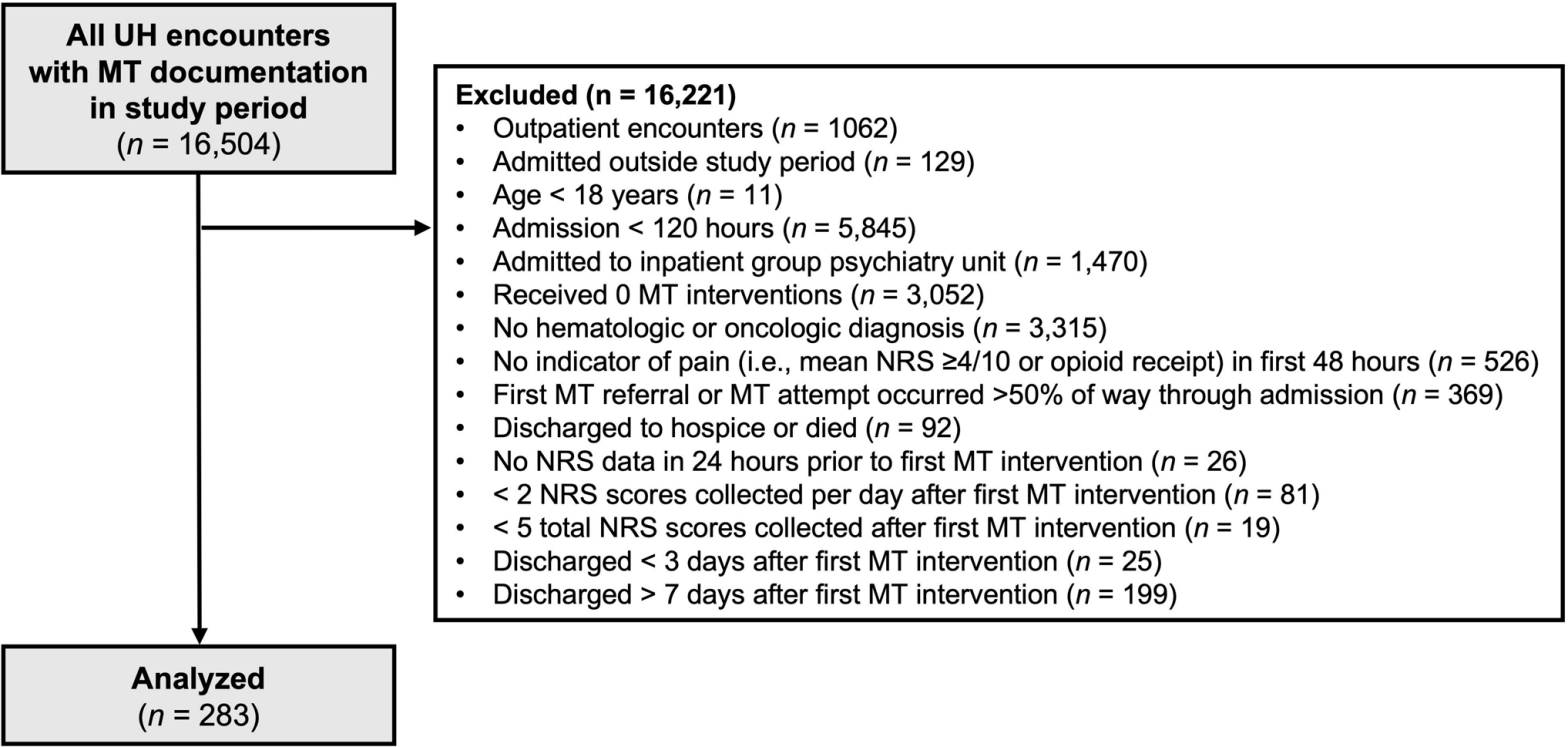
Flowchart of MT Encounter Selection. Abbreviations: MT, music therapy; NRS, numeric rating scale

A flowchart of MT encounter selection is provided in Fig. 1. The final sample included 283 hospital admissions. MT interventions were delivered by 26 different MT clinicians and documented in the EHR. Interventions were provided to patients (mean age 55.92 ± 19.40 years) who were predominantly female (67.1%), non-Hispanic (98.2%), and coded as White (54.1%) or Black/African American (44.5%) within the EHR. Of these 283 hospital admissions, 122 (43.1%) were among patients who received 1, 89 (31.4%) were among patients who received 2, and 72 (25.4%) were among patients who received ≥ 3 MT interventions. Among the 72 hospital admissions where patients received ≥ 3 MT interventions, the exposure distribution was as follows: 50 (69.4%) received 3 MT interventions, 14 (19.4%) received 4 MT interventions, 6 (8.3%) received 5 MT interventions, and 2 (2.8%) received 6 MT interventions. MT interventions were delivered across 10 distinct medical centers, including 2 academic and 8 community medical centers with a median (interquartile range [IQR]) length of stay of 8 (6–11) days. Of 283 admissions, 73 (25.8%) were among patients with SCD. Common oncologic diagnoses included neoplasms of ill-defined, other secondary and unspecified sites (29.3%), digestive organs (12.7%), respiratory and intrathoracic organs (12.4%), breast (7.4%), and female genital organs (7.4%). Of all encounters, 185 (65.4%) included MSUD diagnoses, with the most common being anxiety disorders (44.5%), depressive disorders (42.0%), and opioid-related disorders (21.6%).

### Comparisons across MT exposure groups

Table 1 summarizes selected demographic and clinical characteristics between the three MT exposure groups. There were no meaningful between-group differences observed in most covariates including demographics, social drivers of health, when MT was initiated during patients’ hospital admissions, SCD prevalence, MSUD conditions, neoplasm diagnoses, most major diagnostic clusters (MDC), baseline pain intensity, overall opioid exposure, Elixhauser comorbidities, intensive care unit stays, other psychosocial services provided, or length of stay. Besides total MT intervention exposure (*p* < 0.001), the only other statistically detectable between-group differences (≥ 3 vs. 2 vs. 1 MT intervention[s]) were in the presence of gastrointestinal/hepatic conditions (6.9% vs. 9.0% vs. 19.7%, *p* = 0.015), pulmonary circulation disorders (20.8% vs. 29.2% vs. 13.1%, *p* = 0.016), and the number of days admitted to the hospital between first MT intervention and discharge (6.18 ± 1.69 vs. 5.15 ± 1.57 vs. 4.84 ± 1.51, *p* < 0.001).

**Table 1:** Demographics, Clinical, and MT Utilization Characteristics

Variable	1 MT intervention *N* = 122	2 MT interventions *N* = 89	≥ 3 MT interventions *N* = 72	*p*-value
**Total MT interventions, range**	1.00—1.00	2.00—2.00	3.00—6.00	< 0.001
**% through LOS when MT initiated**	25.25 ± 13.73	24.66 ± 12.90	22.87 ± 12.68	0.416
**Age**	57.76 ± 18.19	54.42 ± 21.19	54.65 ± 19.07	0.394
**Sex**				0.818
Female	81 (66.4%)	62 (69.7%)	47 (65.3%)	
Male	41 (33.6%)	27 (30.3%)	25 (34.7%)	
**Race**				0.745
White	66 (54.1%)	51 (57.3%)	36 (50.0%)	
Black/African American	55 (45.1%)	37 (41.6%)	34 (47.2%)	
Other race	1 (0.8%)	1 (1.1%)	2 (2.8%)	
**Ethnicity**				0.613
Non-Hispanic	121 (99.2%)	87 (97.8%)	70 (97.2%)	
Hispanic/Other	1 (0.8%)	2 (2.2%)	2 (2.8%)	
**Marital status**				0.253
Single	48 (39.3%)	47 (52.8%)	34 (47.2%)	
Married/Life partner	42 (34.4%)	22 (24.7%)	23 (31.9%)	
Widowed	14 (11.5%)	14 (15.7%)	8 (11.1%)	
Divorced/Separated	18 (14.8%)	6 (6.7%)	7 (9.7%)	
**Primary insurance**				0.940
Medicare	69 (56.6%)	45 (50.6%)	38 (52.8%)	
Medicaid	36 (29.5%)	31 (34.8%)	22 (30.6%)	
Private	16 (13.1%)	12 (13.5%)	12 (16.7%)	
Other	1 (0.8%)	1 (1.1%)	0 (0.0%)	
**Mean neighborhood med income**	55,844.15	54,795.38	52,565.72	0.979
**Sickle cell disease**	27 (22.1%)	24 (27.0%)	22 (30.6%)	0.412
**Neoplasm diagnoses**				
Ill-defined, unspecified sites	36 (29.5%)	24 (27.0%)	23 (31.9%)	0.787
Malignant hematology	17 (13.9%)	9 (10.1%)	11 (15.3%)	0.584
Digestive organs	15 (12.3%)	14 (15.7%)	7 (9.7%)	0.515
Respiratory/intrathoracic organs	20 (16.4%)	6 (6.7%)	9 (12.5%)	0.109
Breast	7 (5.7%)	7 (7.9%)	7 (9.7%)	0.582
Female genital organs	10 (8.2%)	7 (7.9%)	4 (5.6%)	0.780
Lip, oral cavity and pharynx	3 (2.5%)	3 (3.4%)	3 (4.2%)	0.763
**Length of stay, median (IQR)**	8.00 (6.00, 10.00)	8.00 (6.00, 10.00)	9.00 (7.00, 11.50)	0.056
**Days adm after MT intervention 1**	4.84 ± 1.51	5.15 ± 1.57	6.18 ± 1.69	< 0.001
**van Walraven Elixhauser weight**	16.88 ± 12.24	15.64 ± 11.05	17.40 ± 12.02	0.706
**Pulmonary circulation disorders**	16 (13.1%)	26 (29.2%)	15 (20.8%)	0.016
**GI/hepatic conditions**	24 (19.7%)	8 (9.0%)	5 (6.9%)	0.015
**MSUD**				
Any MSUD	78 (63.9%)	59 (66.3%)	48 (66.7%)	0.906
Anxiety	52 (42.6%)	40 (44.9%)	34 (47.2%)	0.820
Depression	56 (45.9%)	32 (36.0%)	31 (43.1%)	0.345
Opioid-related disorders	27 (22.1%)	19 (21.3%)	15 (20.8%)	0.976
Trauma/stress-related disorders	15 (12.3%)	10 (11.2%)	8 (11.1%)	0.959
**Baseline measures**				
Pain NRS	5.42 ± 2.93	5.52 ± 2.95	5.10 ± 3.06	0.742
Log-transformed MME	3.39 ± 2.01	3.21 ± 1.96	3.81 ± 1.99	0.146

Continuous variables reported as mean ± standard deviation except where indicated. Categorical variables reported as *n* (%). Comparisons were conducted using Kruskal–Wallis rank sum test, Pearson’s Chi-squared test, and Fisher’s exact test. *adm* admitted, *GI *gastrointestinal, *IQR* interquartile range, *LOS* length of stay, *med* median, *MT* music therapy, *MSUD* mental health and/or substance use disorder, *MME* morphine milligram equivalents, *NRS* numeric rating scale, *SD* standard deviation

### Longitudinal analysis

**Fig. 2:**
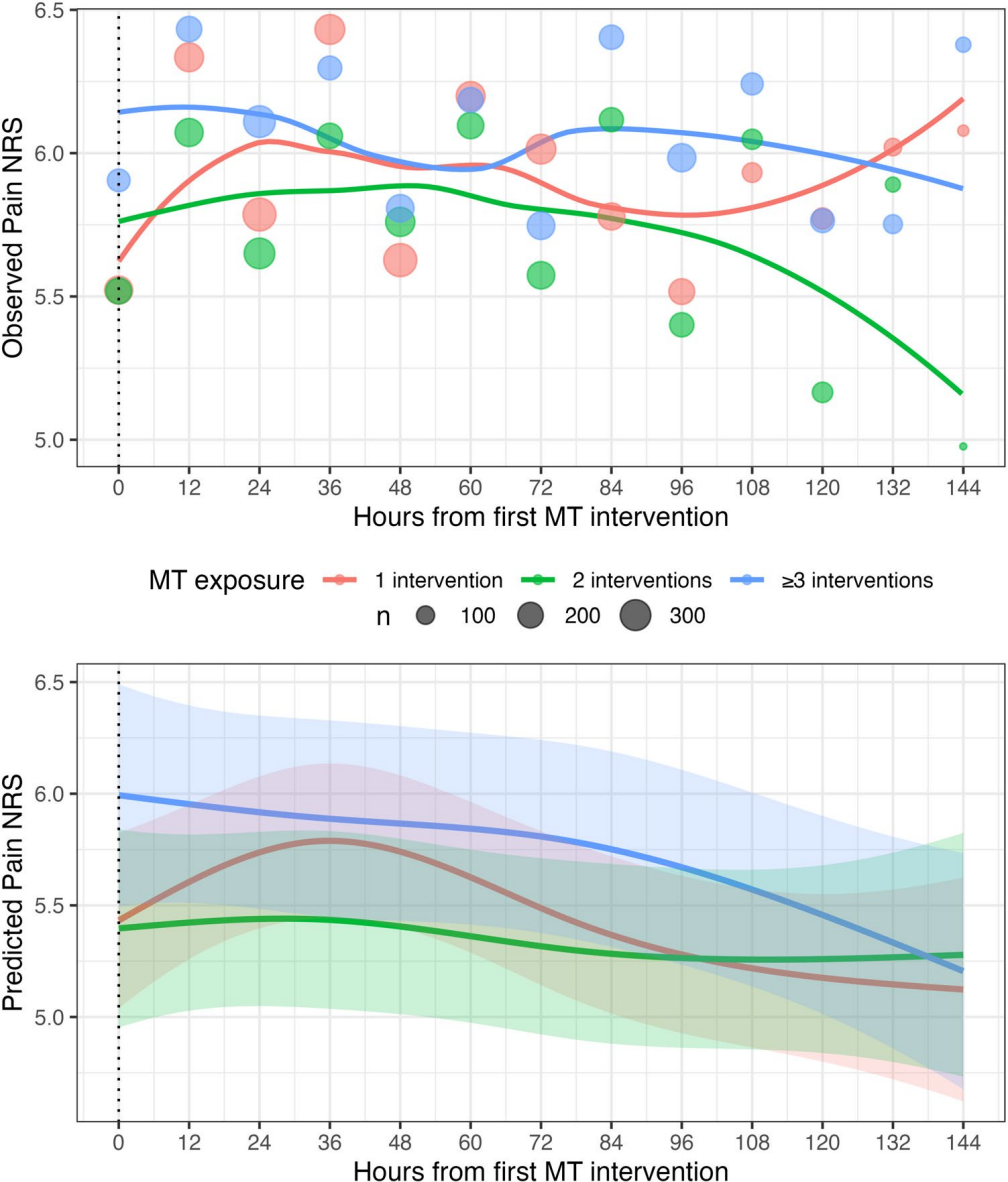
Plot of Longitudinal Pain NRS Outcome Over Time. The top panel (Observed Pain NRS) displays the observed mean pain scores on the numerical rating scale from the time of the first MT intervention up to 144 h following the first MT intervention. Individual circles represent observed mean pain scores at specific time intervals. The size of each circle corresponds to the sample size (n) of available NRS data at that time point. Trend lines represent locally estimated scatterplot smoothing (LOESS) curves of the general trend for patients receiving 1 intervention (red), 2 interventions (green), and ≥ 3 interventions (blue). The bottom panel (Predicted Pain NRS) shows the predicted pain scores derived from the lmer Spline model. The solid lines represent the predicted mean pain trajectory for each MT exposure group. Shaded areas indicate the 95% confidence intervals for the predictions, reflecting the uncertainty in the model over time. Abbreviations: MT, music therapy; NRS, numeric rating scale

**Fig. 3:**
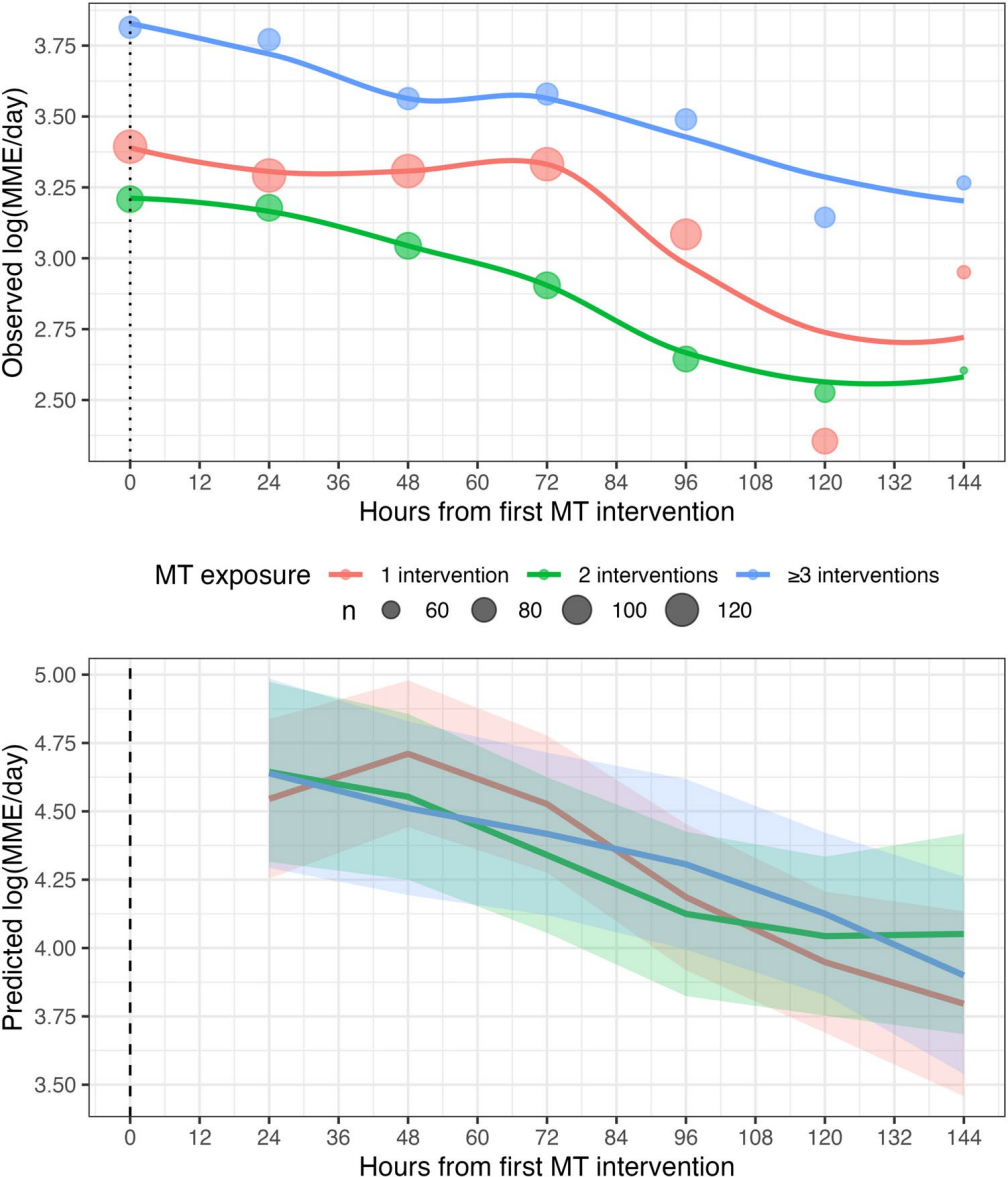
Plot of Longitudinal MME Outcome Over Time. The top panel (Observed log (MME/day)) displays the observed mean log-transformed MME scores from the 24 h prior to the first MT intervention up to 144 h following the first MT intervention. Individual circles represent observed mean log-transformed MME values at specific time intervals. The size of each circle corresponds to the sample size (n) of available MME data at that time point. Trend lines represent locally estimated scatterplot smoothing (LOESS) curves of the general trend for patients receiving 1 intervention (red), 2 interventions (green), and ≥ 3 interventions (blue). The bottom panel (Predicted log (MME/day)) shows the predicted log-transformed MME values derived from the lmer Spline model. The solid lines represent the predicted mean log-transformed MME trajectory for each MT exposure group. Shaded areas indicate the 95% confidence intervals for the predictions, reflecting the uncertainty in the model over time. Differences in baseline values between the observed and predicted trajectories are due to the lmer Spline model accounting for baseline differences in MME administered prior to the first MT intervention. Abbreviations: MT, music therapy; MME, morphine milligram equivalents; NRS, numeric rating scale

Plots summarizing the observed and predicted (within the Spline models) distribution of longitudinal outcomes over time between the three MT exposure groups are provided in Figs. 2 and 3. Individual circles in the top panels represent observed means at specific time intervals. The size of each circle corresponds to the sample size (n) of available outcome data at that time point. Coefficients from the Linear time models are summarized in Table 2. ANOVA tests on each series of models demonstrated no meaningful MT exposure by time effect on pain NRS (Linear time model *p* = 0.158, Spline model *p* = 0.131) or MME/day (Linear time model *p* = 0.570, Spline model *p* = 0.118).

**Table 2:** Results from Linear Time Mixed Effects Models

Effect	Estimate and 95% CI	*p*-value
**Pain model**		
Intercept	1.478 [0.794, 2.162]	< 0.001
Baseline difference 2 MT vs. 1 MT intervention	−0.307 [−0.776, 0.163]	0.200
Baseline difference ≥ 3 MT vs. 1 MT intervention	0.300 [−0.198, 0.799]	0.237
Time (hours)	−0.004 [−0.006, −0.002]	< 0.001
Baseline pain NRS	0.667 [0.602, 0.733]	< 0.001
Length of stay (hours)	0.001 [−0.001, 0.003]	0.550
MT exposure by time (2 vs. 1 MT intervention)	0.002 [−0.001, 0.006]	0.148
MT exposure by time (≥ 3 vs. 1 MT intervention)	−0.001 [−0.004, 0.002]	0.685
**MME model**		
Intercept	0.433 [−0.023, 0.890]	0.062
Baseline difference 2 MT vs. 1 MT intervention	−0.120 [−0.466, 0.227]	0.497
Baseline difference ≥ 3 MT vs. 1 MT intervention	−0.100 [−0.467, 0.267]	0.594
Time (24-h periods)	−0.174 [−0.245, −0.103]	< 0.001
log-transformed baseline MME	0.856 [0.797, 0.915]	< 0.001
Length of stay (hours)	0.001 [0.000, 0.002]	0.117
MT exposure by time (2 vs. 1 MT intervention)	0.030 [−0.041, 0.101]	0.405
MT exposure by time (≥ 3 vs. 1 MT intervention)	0.035 [−0.037, 0.106]	0.339

Linear mixed effects models were fit without nonlinear splines to examine exposure by time effects on longitudinal outcomes after accounting for baseline outcome measures, length of stay (in hours), and random music therapist effects. For the MME model, MME/day outcome measures were quantified within 24-h periods relative to the first MT intervention and log-transformed. Both linear mixed effects models were fit using the lmer function from the “lme4” package. *CI* confidence interval, *MME* morphine milligram equivalents, *MT* music therapy, *NRS* numeric rating scale

## Discussion

The purpose of this study was to determine if there is a dose–response relationship between the number of MT interventions provided and longitudinal changes in pain intensity and MME measured from the time of first MT intervention until 144 h following the first MT intervention. Though this study did not find evidence to support a dose-effectiveness response with MT, methods applied within this analysis may be well-suited to future examinations of longitudinal effects of other inpatient integrative oncology (or non-oncology) programs.

Our findings are in stark contrast to other studies examining dose response effects of MT in other patient populations. A 2009 systematic review and meta-analysis of RCTs, controlled clinical trials, and uncontrolled studies among people (inpatient and outpatient) with serious mental disorders (e.g., psychosis, borderline personality disorder, bipolar disorder) examining dose–response relationships with MT found meaningful dose–effect relationships for general, negative, and depressive symptoms, as well as functioning, with explained variance ranging from 73 to 78%. Small effect sizes for these outcomes were achieved after 3–10 interventions and large effects after 16–51 interventions [15].

Our results also contrast with evidence from other integrative oncology modalities. A pragmatic non-randomized prospective study comparing participation in ≥ 4 outpatient integrative oncology treatments (mean treatments: 6.9 ± 1.9 that included guidance on herbs and supplements, acupuncture, reflexology, movement therapies [e.g., Qigong], guided imagery, MT, and/or spiritual care) to ≤ 3 (mean 2.4 ± 1.4) among 439 patients with cancer undergoing adjuvant/neo-adjuvant chemotherapy for localized cancer (stages I – III) found that patients in the higher adherence (i.e., higher dose) group reported greater improvement on ESAS sleep from baseline to 6 weeks (*p* = 0.044) [5]. A 2021 RCT examining outpatient yoga practice among women with metastatic cancer found that among women randomized to yoga, a dose–response relationship was found between yoga practice duration and daily pain. When patients had spent relatively more time practicing yoga across two consecutive days, they were more likely to experience lower pain on the next day [11].

Several factors related to MT intervention delivery, data collection, patients’ pre-hospitalization opioid exposure, and healthcare professional behavior may explain why no dose–response effect was observed. First, 2 or 3–6 MT interventions may be insufficient doses relative to 1 MT intervention or no intervention at all for influencing longitudinal outcomes during a hospital admission. Given that many cancer centers only have 1–2 music therapists and music therapists’ caseloads are often so high that they are unable to see patients every day, it is challenging for MT to be delivered at an optimal dose (e.g., every day). Prior studies demonstrating reduction in opioid utilization in response to MT featured higher doses such as 10 weekly outpatient sessions among patients with advanced cancer [8]. Second, there was a high prevalence of opioid-related disorders within this sample (21.6%). Thus, many patients may have had pre-existing opioid utilization patterns that would be challenging to change over the course of a hospital admission.

Third, changing the course of a patient’s opioid administration requires actions across healthcare organizations. Specifically, studies that have demonstrated success in reducing opioid exposure have involved multifaceted organizational interventions [22, 28] including (1) implementing new guidelines for opioid administration; (2) educating physicians, nurses, pharmacists, and other allied health professionals on nonpharmacologic analgesic approaches; (3) changing EHR workflow; (4) audit and feedback; (5) educating patients; and (6) electronically monitoring patients’ pain. Finally, although the MT exposure-by-time interaction did not provide strong statistical evidence of an association and most measured baseline characteristics were similar across the three MT exposure groups, the observed outcome trajectories in Figs. 2 and 3 suggest the possibility of unmeasured between-group differences. For example, patients receiving ≥ 3 MT sessions may represent a subgroup with more persistent or difficult-to-manage symptoms, which could contribute to the relatively stable symptom trajectories observed in this group compared with those receiving fewer MT sessions. Given these factors, more research is needed with a greater exposure gap (e.g., an RCT comparing daily MT exposure to 1 MT exposure) and controlled organization-level actions (e.g., early MT referral, increased symptom monitoring, and provider education) to investigate this dose–response effect.

Finally, given that chronic pain is highly prevalent among patients with cancer [19] and patients with SCD [9, 40], pain intensity may be especially challenging to modulate as an outcome over the course of a hospital admission within this population, especially when measured using a single item NRS. Prior studies have demonstrated that individuals with chronic pain may interpret pain intensity scales differently than people with acute pain [42]. Though an efficient and valid tool for measuring pain intensity in the hospital [20, 27], the NRS is not well-suited toward measuring pain’s impact on individuals’ daily lives, interference with daily activities, or perceived ability to cope despite pain [2]. Engaging in MT may reduce pain interference among individuals with chronic pain related to cancer [7] or SCD [35] without necessarily affecting individuals' perceived pain intensity [2, 33]. However, pain interference is not a routinely measured outcome during hospital admissions. Thus, future studies examining dose–response relationships may want to consider evaluating responses among populations who develop high pain intensity during the hospital admission (e.g., post-surgery) rather than presenting to the hospital with chronically high pain intensity due to hematologic and/or oncologic conditions.

Strengths of this study include (1) a large sample size across 10 medical centers; (2) high Black/African American representation (44.5%) as compared to other MT studies among patients with cancer [6]; and (3) a novel approach to using EHR data to measure MT’s real-world longitudinal effectiveness. Important limitations include the use of single-item NRS measures rather than more comprehensive pain instruments, a lack of control over the frequency or accuracy of nurses’ collection of NRS measures, and a lack of outcome data on other clinically-relevant measures for which MT has been shown to be effective (e.g., stress, anxiety, depression) [10, 39]. Pain intensity and opioid utilization were selected because they are consistently documented in the inpatient EHR and therefore available for longitudinal analysis. However, these measures may only capture a subset of MT’s potential clinical impact. In inpatient settings, psychosocial outcomes addressed by music therapists are not routinely collected. Thus, the present findings should be interpreted within the constraints of EHR-derived outcomes and may underestimate broader therapeutic effects among hospitalized patients with hematologic/oncologic conditions. Prospective studies incorporating multidimensional symptom and psychosocial outcome measures may provide a more comprehensive evaluation of MT’s impact.

Given that MT exposure in this retrospective study was determined by clinical referral patterns rather than randomization, patients who received multiple MT sessions likely differed systematically from those receiving fewer sessions or no sessions at all. A 2016 EHR review of 83,677 hospital admission found that healthcare professionals were more likely to refer patients to inpatient integrative medicine if they had hard-to-manage symptoms and/or did not respond to conventional therapies [16]. Our recent retrospective study demonstrated that patients with MSUD who underwent surgery and received 2–10 MT interventions were more medically complex that those who did not receive MT [36]. Although models adjusted for baseline pain intensity, baseline opioid utilization, and length of stay, unmeasured factors such as clinician referral preferences, patient interest in MT, and clinical complexity may have influenced both MT exposure and outcomes. MT exposure was treated as a categorical variable based on number of interventions provided and did not account for variations in MT interventions delivered across sessions. This is notable as our recent analysis of 2,039 MT interventions delivered across the UH health system found that MT interventions involving singing, active instrument play, and relaxation/imagery may be more effective for reducing pain intensity on the NRS than interventions only involving live or recorded music [34]. Finally, as with many retrospective EHR studies, demographic data including sex, race, and ethnicity were extracted exactly as they were entered into the EHR by HCPs and may not have reflected the gender, racial, and/or ethnic identities of the patients included in this study [21].

## Conclusion

Among patients admitted with hematologic/oncologic conditions, receiving greater MT intervention exposure does not appear to reduce pain intensity or opioid exposure over time relative to receiving 1 MT intervention. Methods applied within this analysis may be well-suited to examining longitudinal effects of other inpatient integrative oncology programs. Future evaluations of such programs should consider collecting more robust data on outcomes that may be more sensitive to intervention (e.g., stress, anxiety, fatigue, and pain interference) and increasing the dose (e.g., daily administration) of integrative therapies to have a greater impact on outcomes over time.

## Data Availability

The datasets generated and/or analyzed during the current study are not publicly available due to privacy restrictions as the databases contain information that could compromise the privacy of research participants. However, the de-identified datasets are available from the corresponding author on reasonable request.
